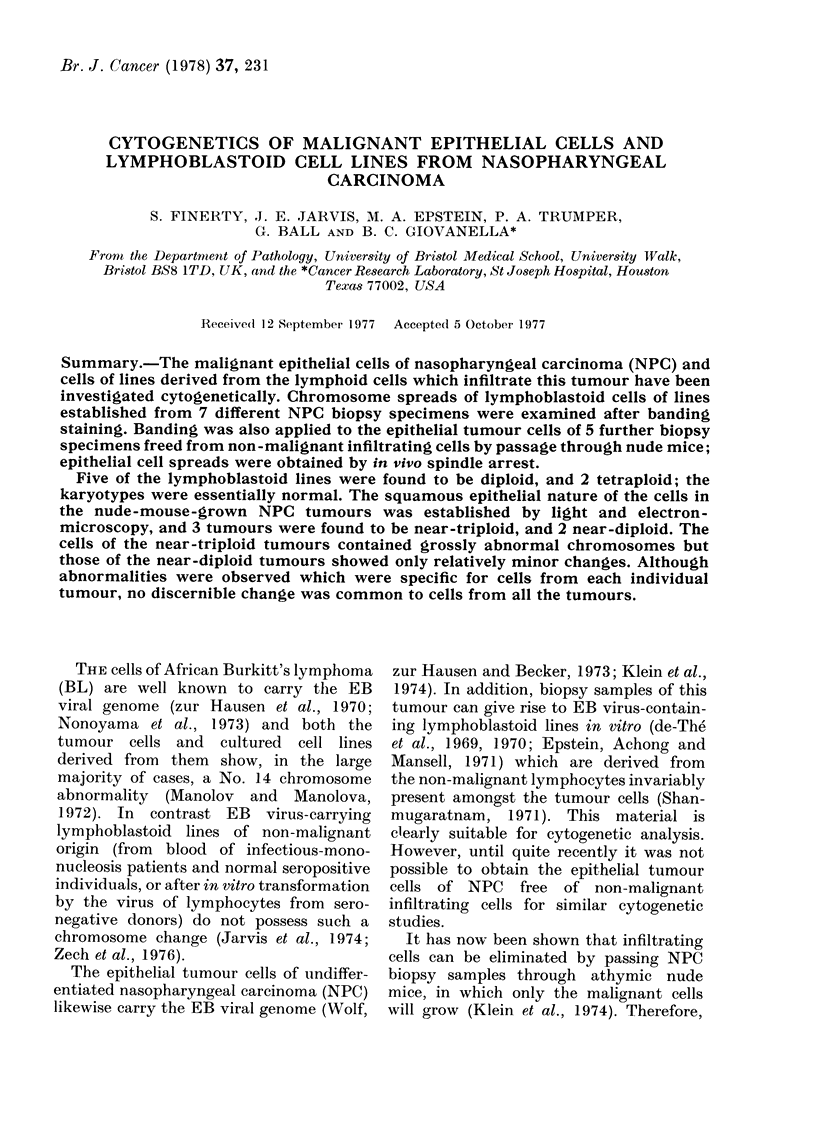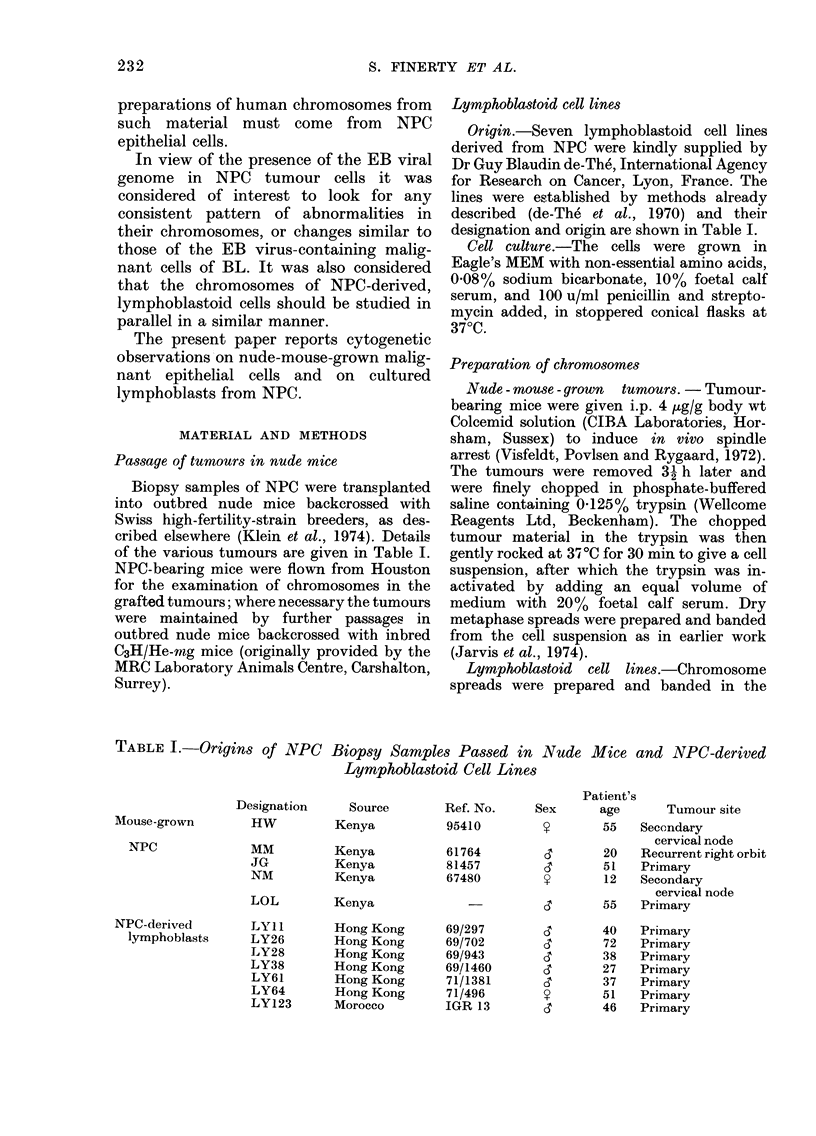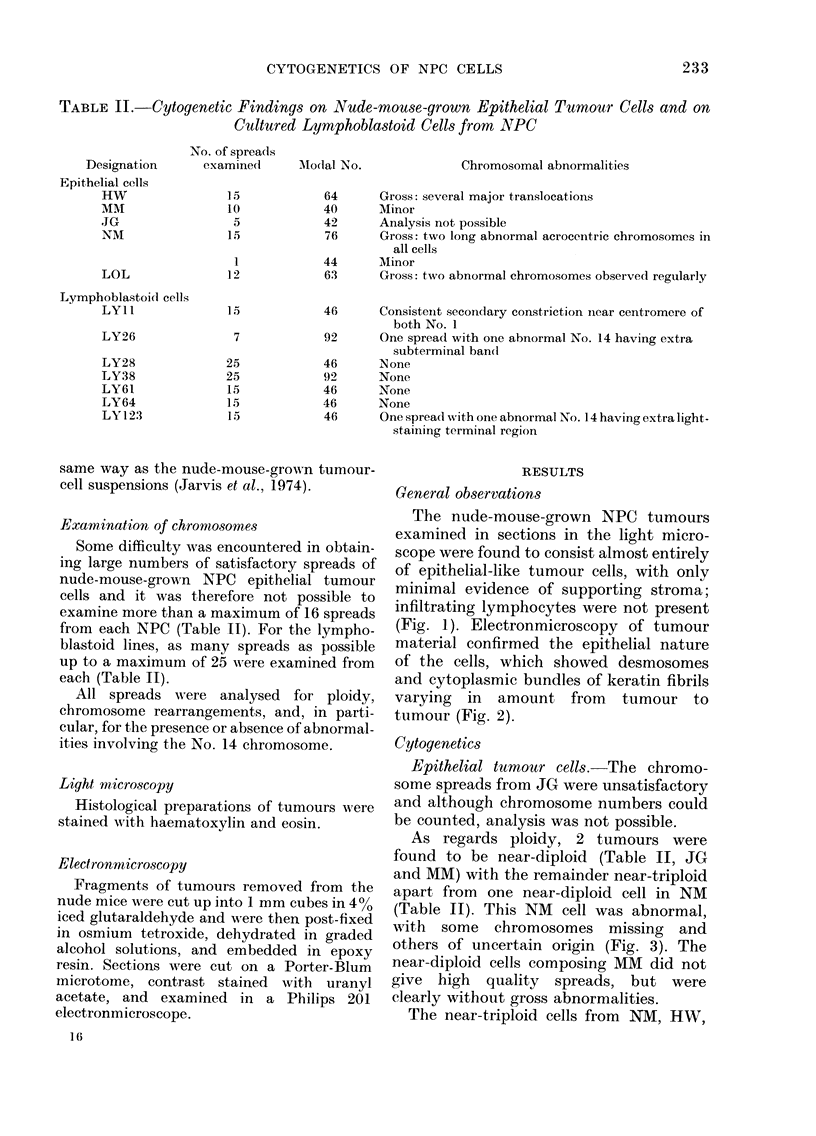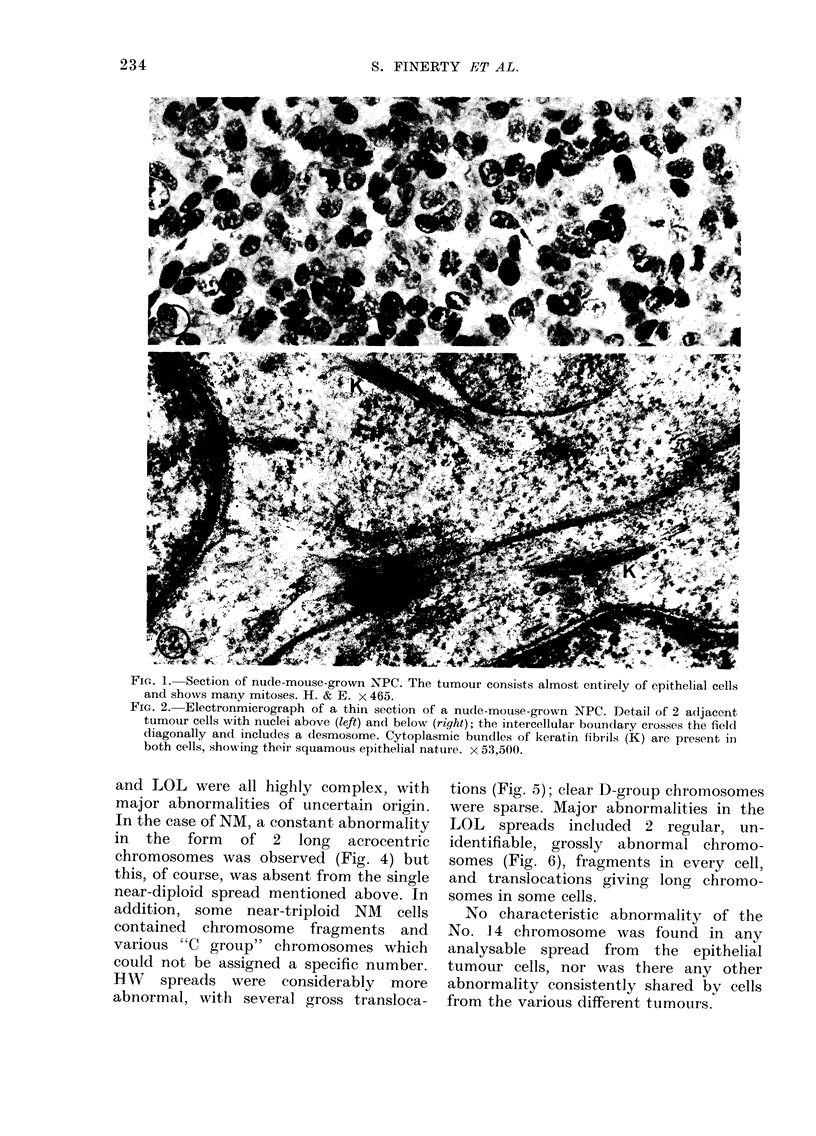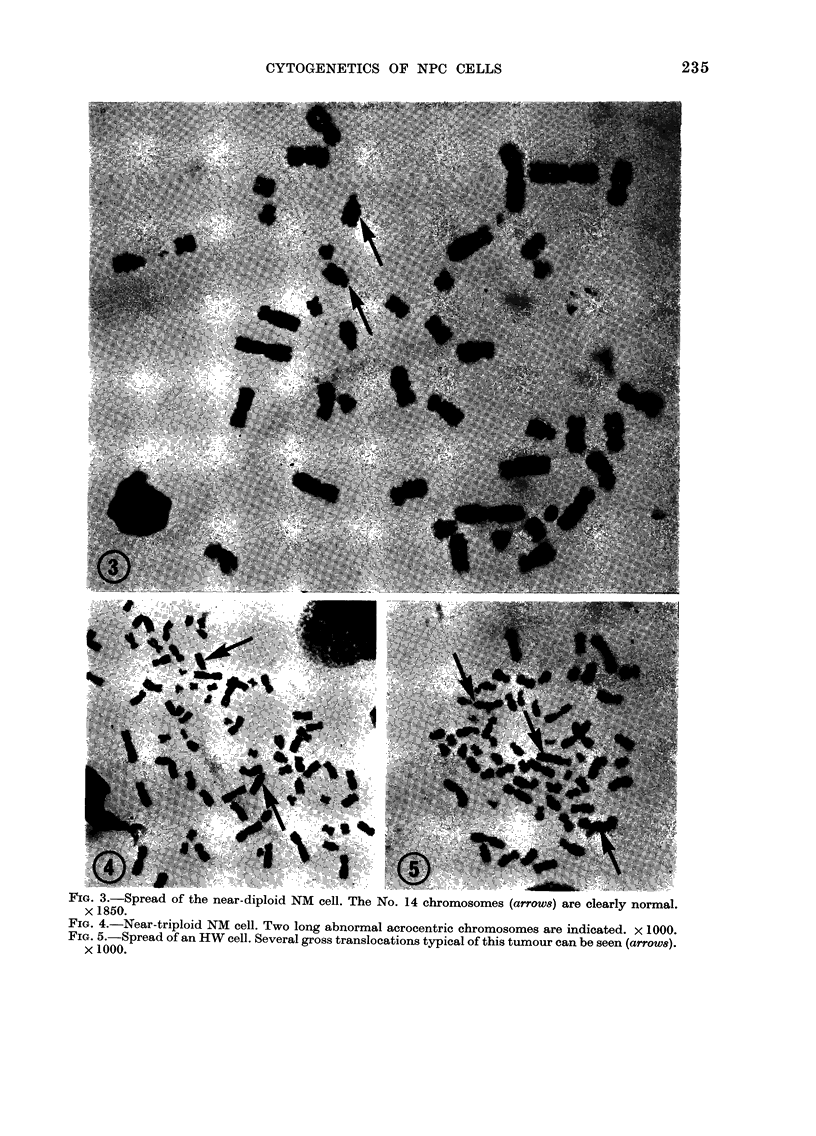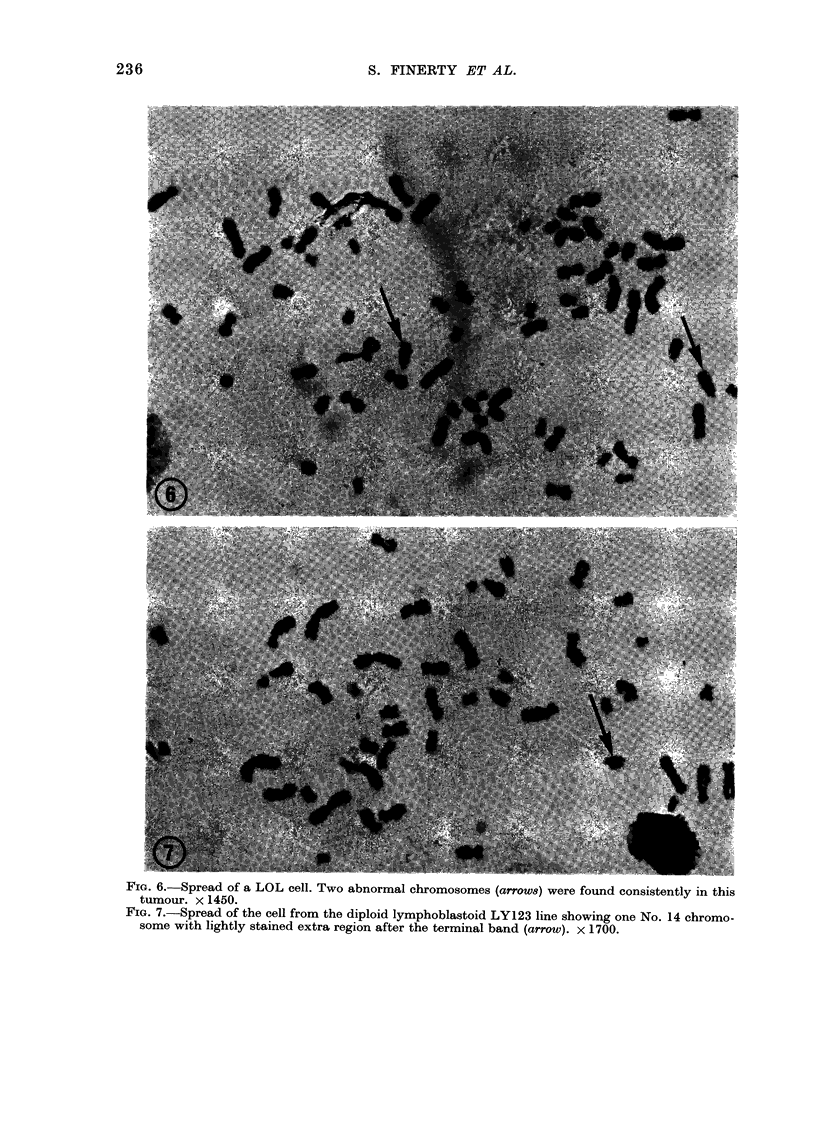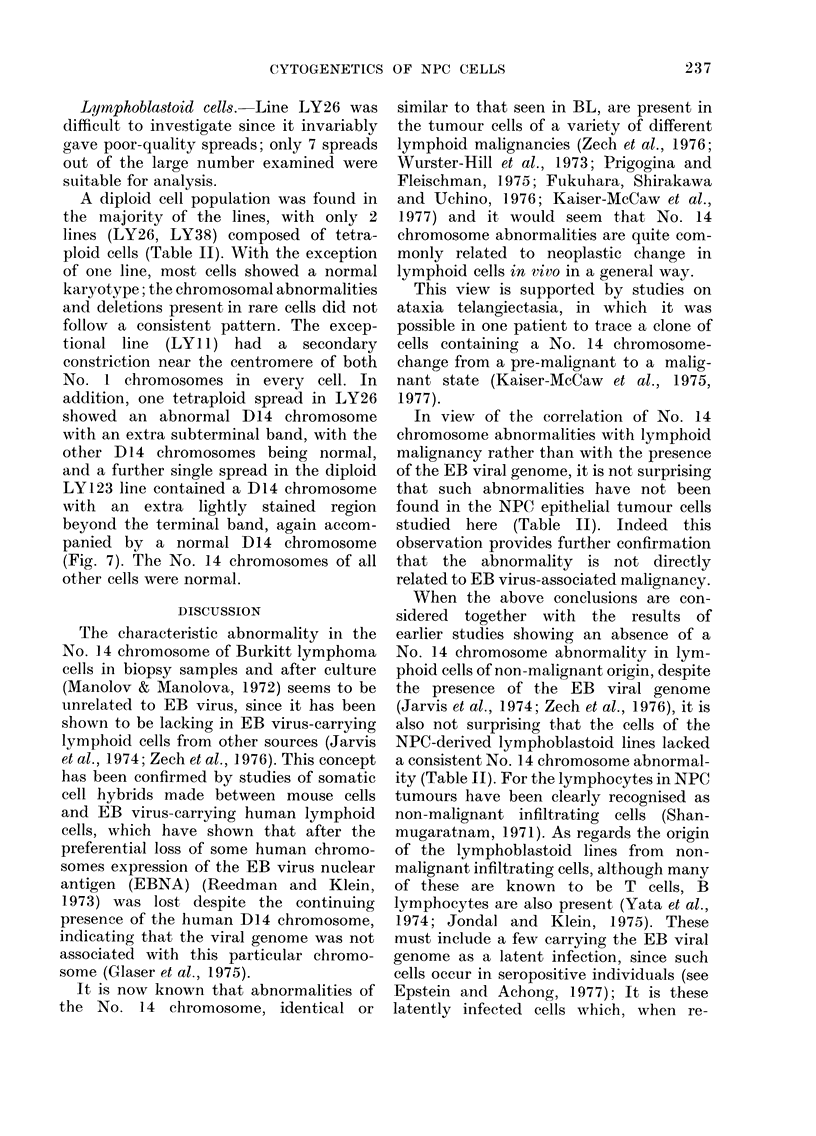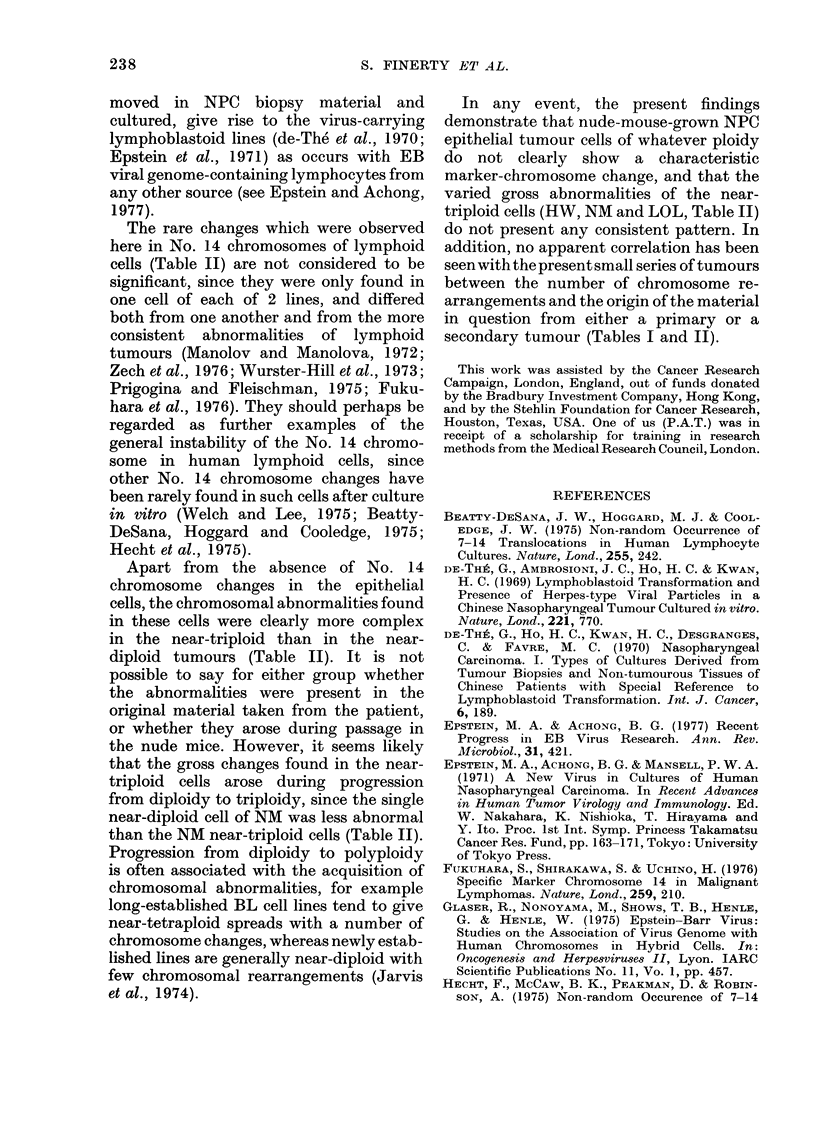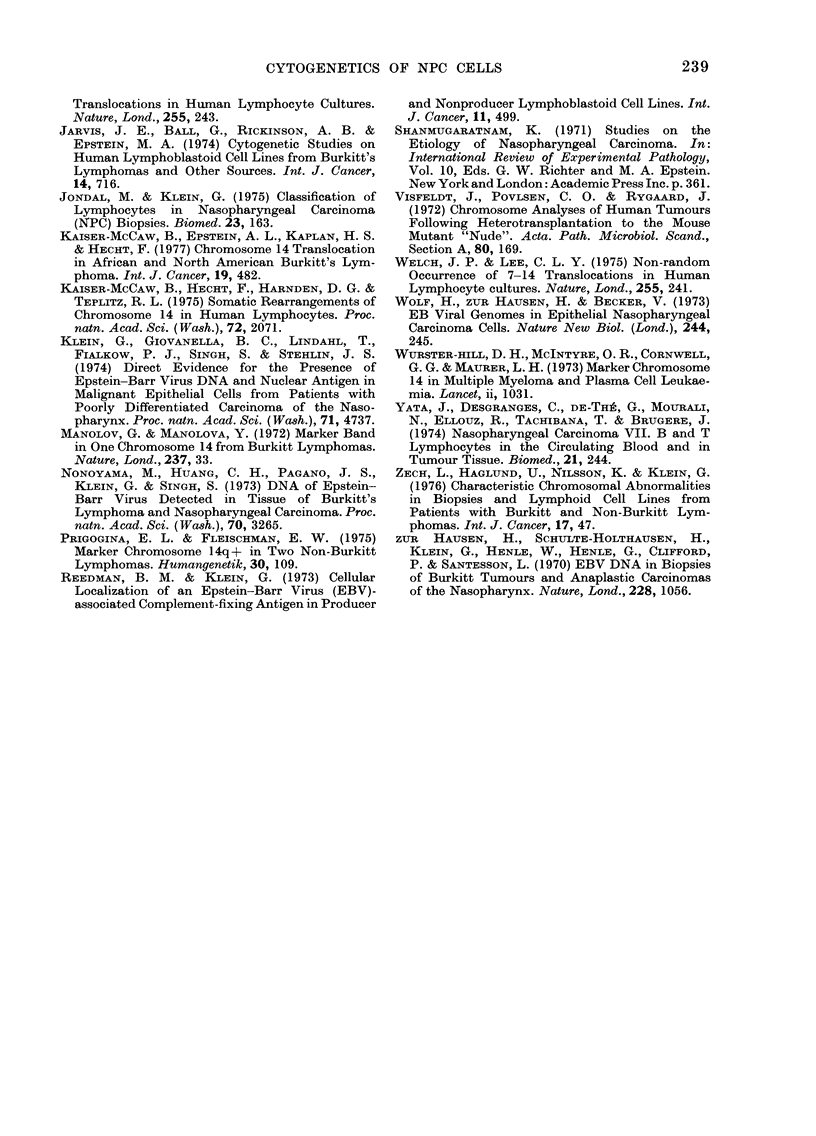# Cytogenetics of malignant epithelial cells and lymphoblastoid cell lines from nasopharyngeal carcinoma.

**DOI:** 10.1038/bjc.1978.31

**Published:** 1978-02

**Authors:** S. Finerty, J. E. Jarvis, M. A. Epstein, P. A. Trumper, G. Ball, B. C. Giovanella

## Abstract

**Images:**


*Br. J. Cancer* (1978) **37**, 231

### CYTOGENETICS OF MALIGNANT EPITHELIAL CELLS AND LYMPHOBLASTOID CELL LINES FROM NASOPHARYNGEAL CARCINOMA

S. FINERTY, J. E. JARVIS, M. A. EPSTEIN, P. A. TRUMPER, G. BALL AND B. C. GIOVANELLA*

*From the Department of Pathology, University of Bristol Medical School, University Walk, Bristol BS8 1TD, UK, and the *Cancer Research Laboratory, St Joseph Hospital, Houston Texas 77002, USA*

Received 12 September 1977 Accepted 5 October 1977

**Summary.—The malignant epithelial cells of nasopharyngeal carcinoma (NPC) and cells of lines derived from the lymphoid cells which infiltrate this tumour have been investigated cytogenetically. Chromosome spreads of lymphoblastoid cells of lines established from 7 different NPC biopsy specimens were examined after banding staining. Banding was also applied to the epithelial tumour cells of 5 further biopsy specimens freed from non-malignant infiltrating cells by passage through nude mice; epithelial cell spreads were obtained by *in vivo* spindle arrest.**

**Five of the lymphoblastoid lines were found to be diploid, and 2 tetraploid; the karyotypes were essentially normal. The squamous epithelial nature of the cells in the nude-mouse-grown NPC tumours was established by light and electron-microscopy, and 3 tumours were found to be near-triploid, and 2 near-diploid. The cells of the near-triploid tumours contained grossly abnormal chromosomes but those of the near-diploid tumours showed only relatively minor changes. Although abnormalities were observed which were specific for cells from each individual tumour, no discernible change was common to cells from all the tumours.**

THE cells of African Burkitt's lymphoma (BL) are well known to carry the EB viral genome (zur Hausen *et al.*, 1970; Nonoyama *et al.*, 1973) and both the tumour cells and cultured cell lines derived from them show, in the large majority of cases, a No. 14 chromosome abnormality (Manolov and Manolova, 1972). In contrast EB virus-carrying lymphoblastoid lines of non-malignant origin (from blood of infectious-mononucleosis patients and normal seropositive individuals, or after *in vitro* transformation by the virus of lymphocytes from seronegative donors) do not possess such a chromosome change (Jarvis *et al.*, 1974; Zech *et al.*, 1976).

The epithelial tumour cells of undifferentiated nasopharyngeal carcinoma (NPC) likewise carry the EB viral genome (Wolf, zur Hausen and Becker, 1973; Klein *et al.*, 1974). In addition, biopsy samples of this tumour can give rise to EB virus-containing lymphoblastoid lines *in vitro* (de-Thé *et al.*, 1969, 1970; Epstein, Achong and Mansell, 1971) which are derived from the non-malignant lymphocytes invariably present amongst the tumour cells (Shanmugaratnam, 1971). This material is clearly suitable for cytogenetic analysis. However, until quite recently it was not possible to obtain the epithelial tumour cells of NPC free of non-malignant infiltrating cells for similar cytogenetic studies.

It has now been shown that infiltrating cells can be eliminated by passing NPC biopsy samples through athymic nude mice, in which only the malignant cells will grow (Klein *et al.*, 1974). Therefore, preparations of human chromosomes from such material must come from NPC epithelial cells.

In view of the presence of the EB viral genome in NPC tumour cells it was considered of interest to look for any consistent pattern of abnormalities in their chromosomes, or changes similar to those of the EB virus-containing malignant cells of BL. It was also considered that the chromosomes of NPC-derived, lymphoblastoid cells should be studied in parallel in a similar manner.

The present paper reports cytogenetic observations on nude-mouse-grown malignant epithelial cells and on cultured lymphoblasts from NPC.

### MATERIAL AND METHODS

### *Passage of tumours in nude mice*

Biopsy samples of NPC were transplanted into outbred nude mice backcrossed with Swiss high-fertility-strain breeders, as described elsewhere (Klein *et al.*, 1974). Details of the various tumours are given in Table I. NPC-bearing mice were flown from Houston for the examination of chromosomes in the grafted tumours; where necessary the tumours were maintained by further passages in outbred nude mice backcrossed with inbred C3H/He-*mg* mice (originally provided by the MRC Laboratory Animals Centre, Carshalton, Surrey).

### *Lymphoblastoid cell lines*

*Origin.*—Seven lymphoblastoid cell lines derived from NPC were kindly supplied by Dr Guy Blaudin de-Thé, International Agency for Research on Cancer, Lyon, France. The lines were established by methods already described (de-Thé *et al.*, 1970) and their designation and origin are shown in Table I.

*Cell culture.*—The cells were grown in Eagle's MEM with non-essential amino acids, 0·08% sodium bicarbonate, 10% foetal calf serum, and 100 u/ml penicillin and streptomycin added, in stoppered conical flasks at 37°C.

### *Preparation of chromosomes*

*Nude-mouse-grown tumours.* — Tumour-bearing mice were given i.p. 4 $\mu$g/g body wt Colcemid solution (CIBA Laboratories, Horsham, Sussex) to induce *in vivo* spindle arrest (Visfeldt, Povlsen and Rygaard, 1972). The tumours were removed $3\frac{1}{2}$ h later and were finely chopped in phosphate-buffered saline containing 0·125% trypsin (Wellcome Reagents Ltd, Beckenham). The chopped tumour material in the trypsin was then gently rocked at 37°C for 30 min to give a cell suspension, after which the trypsin was inactivated by adding an equal volume of medium with 20% foetal calf serum. Dry metaphase spreads were prepared and banded from the cell suspension as in earlier work (Jarvis *et al.*, 1974).

*Lymphoblastoid cell lines.*—Chromosome spreads were prepared and banded in the

TABLE I.—*Origins of NPC Biopsy Samples Passed in Nude Mice and NPC-derived Lymphoblastoid Cell Lines*

| | Designation | Source | Ref. No. | Sex | Patient's age | Tumour site |
|---|---|---|---|---|---|---|
| Mouse-grown NPC | HW | Kenya | 95410 | ♀ | 55 | Secondary cervical node |
| | MM | Kenya | 61764 | ♂ | 20 | Recurrent right orbit |
| | JG | Kenya | 81457 | ♂ | 51 | Primary |
| | NM | Kenya | 67480 | ♀ | 12 | Secondary cervical node |
| | LOL | Kenya | — | ♂ | 55 | Primary |
| NPC-derived lymphoblasts | LY11 | Hong Kong | 69/297 | ♂ | 40 | Primary |
| | LY26 | Hong Kong | 69/702 | ♂ | 72 | Primary |
| | LY28 | Hong Kong | 69/943 | ♂ | 38 | Primary |
| | LY38 | Hong Kong | 69/1460 | ♂ | 27 | Primary |
| | LY61 | Hong Kong | 71/1381 | ♂ | 37 | Primary |
| | LY64 | Hong Kong | 71/496 | ♀ | 51 | Primary |
| | LY123 | Morocco | IGR 13 | ♂ | 46 | Primary |

TABLE II.—*Cytogenetic Findings on Nude-mouse-grown Epithelial Tumour Cells and on Cultured Lymphoblastoid Cells from NPC*

| Designation | No. of spreads examined | Modal No. | Chromosomal abnormalities |
|---|---|---|---|
| Epithelial cells | | | |
| HW | 15 | 64 | Gross: several major translocations |
| MM | 10 | 40 | Minor |
| JG | 5 | 42 | Analysis not possible |
| NM | 15 | 76 | Gross: two long abnormal acrocentric chromosomes in all cells |
| | 1 | 44 | Minor |
| LOL | 12 | 63 | Gross: two abnormal chromosomes observed regularly |
| Lymphoblastoid cells | | | |
| LY11 | 15 | 46 | Consistent secondary constriction near centromere of both No. 1 |
| LY26 | 7 | 92 | One spread with one abnormal No. 14 having extra subterminal band |
| LY28 | 25 | 46 | None |
| LY38 | 25 | 92 | None |
| LY61 | 15 | 46 | None |
| LY64 | 15 | 46 | None |
| LY123 | 15 | 46 | One spread with one abnormal No. 14 having extra light-staining terminal region |

same way as the nude-mouse-grown tumour-cell suspensions (Jarvis *et al.*, 1974).

*Examination of chromosomes*

Some difficulty was encountered in obtaining large numbers of satisfactory spreads of nude-mouse-grown NPC epithelial tumour cells and it was therefore not possible to examine more than a maximum of 16 spreads from each NPC (Table II). For the lymphoblastoid lines, as many spreads as possible up to a maximum of 25 were examined from each (Table II).

All spreads were analysed for ploidy, chromosome rearrangements, and, in particular, for the presence or absence of abnormalities involving the No. 14 chromosome.

*Light microscopy*

Histological preparations of tumours were stained with haematoxylin and eosin.

*Electronmicroscopy*

Fragments of tumours removed from the nude mice were cut up into 1 mm cubes in 4% iced glutaraldehyde and were then post-fixed in osmium tetroxide, dehydrated in graded alcohol solutions, and embedded in epoxy resin. Sections were cut on a Porter-Blum microtome, contrast stained with uranyl acetate, and examined in a Philips 201 electronmicroscope.

RESULTS

*General observations*

The nude-mouse-grown NPC tumours examined in sections in the light microscope were found to consist almost entirely of epithelial-like tumour cells, with only minimal evidence of supporting stroma; infiltrating lymphocytes were not present (Fig. 1). Electronmicroscopy of tumour material confirmed the epithelial nature of the cells, which showed desmosomes and cytoplasmic bundles of keratin fibrils varying in amount from tumour to tumour (Fig. 2).

*Cytogenetics*

*Epithelial tumour cells.*—The chromosome spreads from JG were unsatisfactory and although chromosome numbers could be counted, analysis was not possible.

As regards ploidy, 2 tumours were found to be near-diploid (Table II, JG and MM) with the remainder near-triploid apart from one near-diploid cell in NM (Table II). This NM cell was abnormal, with some chromosomes missing and others of uncertain origin (Fig. 3). The near-diploid cells composing MM did not give high quality spreads, but were clearly without gross abnormalities.

The near-triploid cells from NM, HW,

FIG. 1.—Section of nude-mouse-grown NPC. The tumour consists almost entirely of epithelial cells and shows many mitoses. H. & E. ×465.

FIG. 2.—Electronmicrograph of a thin section of a nude-mouse-grown NPC. Detail of 2 adjacent tumour cells with nuclei above (*left*) and below (*right*); the intercellular boundary crosses the field diagonally and includes a desmosome. Cytoplasmic bundles of keratin fibrils (K) are present in both cells, showing their squamous epithelial nature. ×53,500.

and LOL were all highly complex, with major abnormalities of uncertain origin. In the case of NM, a constant abnormality in the form of 2 long acrocentric chromosomes was observed (Fig. 4) but this, of course, was absent from the single near-diploid spread mentioned above. In addition, some near-triploid NM cells contained chromosome fragments and various "C group" chromosomes which could not be assigned a specific number. HW spreads were considerably more abnormal, with several gross translocations (Fig. 5); clear D-group chromosomes were sparse. Major abnormalities in the LOL spreads included 2 regular, unidentifiable, grossly abnormal chromosomes (Fig. 6), fragments in every cell, and translocations giving long chromosomes in some cells.

No characteristic abnormality of the No. 14 chromosome was found in any analysable spread from the epithelial tumour cells, nor was there any other abnormality consistently shared by cells from the various different tumours.

FIG. 3.—Spread of the near-diploid NM cell. The No. 14 chromosomes (*arrows*) are clearly normal. ×1850.

FIG. 4.—Near-triploid NM cell. Two long abnormal acrocentric chromosomes are indicated. ×1000.

FIG. 5.—Spread of an HW cell. Several gross translocations typical of this tumour can be seen (*arrows*). ×1000.

FIG. 6.—Spread of a LOL cell. Two abnormal chromosomes (*arrows*) were found consistently in this tumour. ×1450.

FIG. 7.—Spread of the cell from the diploid lymphoblastoid LY123 line showing one No. 14 chromosome with lightly stained extra region after the terminal band (*arrow*). ×1700.

*Lymphoblastoid cells*.—Line LY26 was difficult to investigate since it invariably gave poor-quality spreads; only 7 spreads out of the large number examined were suitable for analysis.

A diploid cell population was found in the majority of the lines, with only 2 lines (LY26, LY38) composed of tetraploid cells (Table II). With the exception of one line, most cells showed a normal karyotype; the chromosomal abnormalities and deletions present in rare cells did not follow a consistent pattern. The exceptional line (LY11) had a secondary constriction near the centromere of both No. 1 chromosomes in every cell. In addition, one tetraploid spread in LY26 showed an abnormal D14 chromosome with an extra subterminal band, with the other D14 chromosomes being normal, and a further single spread in the diploid LY123 line contained a D14 chromosome with an extra lightly stained region beyond the terminal band, again accompanied by a normal D14 chromosome (Fig. 7). The No. 14 chromosomes of all other cells were normal.

### DISCUSSION

The characteristic abnormality in the No. 14 chromosome of Burkitt lymphoma cells in biopsy samples and after culture (Manolov & Manolova, 1972) seems to be unrelated to EB virus, since it has been shown to be lacking in EB virus-carrying lymphoid cells from other sources (Jarvis *et al.*, 1974; Zech *et al.*, 1976). This concept has been confirmed by studies of somatic cell hybrids made between mouse cells and EB virus-carrying human lymphoid cells, which have shown that after the preferential loss of some human chromosomes expression of the EB virus nuclear antigen (EBNA) (Reedman and Klein, 1973) was lost despite the continuing presence of the human D14 chromosome, indicating that the viral genome was not associated with this particular chromosome (Glaser *et al.*, 1975).

It is now known that abnormalities of the No. 14 chromosome, identical or similar to that seen in BL, are present in the tumour cells of a variety of different lymphoid malignancies (Zech *et al.*, 1976; Wurster-Hill *et al.*, 1973; Prigogina and Fleischman, 1975; Fukuhara, Shirakawa and Uchino, 1976; Kaiser-McCaw *et al.*, 1977) and it would seem that No. 14 chromosome abnormalities are quite commonly related to neoplastic change in lymphoid cells *in vivo* in a general way.

This view is supported by studies on ataxia telangiectasia, in which it was possible in one patient to trace a clone of cells containing a No. 14 chromosome-change from a pre-malignant to a malignant state (Kaiser-McCaw *et al.*, 1975, 1977).

In view of the correlation of No. 14 chromosome abnormalities with lymphoid malignancy rather than with the presence of the EB viral genome, it is not surprising that such abnormalities have not been found in the NPC epithelial tumour cells studied here (Table II). Indeed this observation provides further confirmation that the abnormality is not directly related to EB virus-associated malignancy.

When the above conclusions are considered together with the results of earlier studies showing an absence of a No. 14 chromosome abnormality in lymphoid cells of non-malignant origin, despite the presence of the EB viral genome (Jarvis *et al.*, 1974; Zech *et al.*, 1976), it is also not surprising that the cells of the NPC-derived lymphoblastoid lines lacked a consistent No. 14 chromosome abnormality (Table II). For the lymphocytes in NPC tumours have been clearly recognised as non-malignant infiltrating cells (Shanmugaratnam, 1971). As regards the origin of the lymphoblastoid lines from non-malignant infiltrating cells, although many of these are known to be T cells, B lymphocytes are also present (Yata *et al.*, 1974; Jondal and Klein, 1975). These must include a few carrying the EB viral genome as a latent infection, since such cells occur in seropositive individuals (see Epstein and Achong, 1977); It is these latently infected cells which, when removed in NPC biopsy material and cultured, give rise to the virus-carrying lymphoblastoid lines (de-Thé *et al.*, 1970; Epstein *et al.*, 1971) as occurs with EB viral genome-containing lymphocytes from any other source (see Epstein and Achong, 1977).

The rare changes which were observed here in No. 14 chromosomes of lymphoid cells (Table II) are not considered to be significant, since they were only found in one cell of each of 2 lines, and differed both from one another and from the more consistent abnormalities of lymphoid tumours (Manolov and Manolova, 1972; Zech *et al.*, 1976; Wurster-Hill *et al.*, 1973; Prigogina and Fleischman, 1975; Fukuhara *et al.*, 1976). They should perhaps be regarded as further examples of the general instability of the No. 14 chromosome in human lymphoid cells, since other No. 14 chromosome changes have been rarely found in such cells after culture *in vitro* (Welch and Lee, 1975; Beatty-DeSana, Hoggard and Cooledge, 1975; Hecht *et al.*, 1975).

Apart from the absence of No. 14 chromosome changes in the epithelial cells, the chromosomal abnormalities found in these cells were clearly more complex in the near-triploid than in the near-diploid tumours (Table II). It is not possible to say for either group whether the abnormalities were present in the original material taken from the patient, or whether they arose during passage in the nude mice. However, it seems likely that the gross changes found in the near-triploid cells arose during progression from diploidy to triploidy, since the single near-diploid cell of NM was less abnormal than the NM near-triploid cells (Table II). Progression from diploidy to polyploidy is often associated with the acquisition of chromosomal abnormalities, for example long-established BL cell lines tend to give near-tetraploid spreads with a number of chromosome changes, whereas newly established lines are generally near-diploid with few chromosomal rearrangements (Jarvis *et al.*, 1974).

In any event, the present findings demonstrate that nude-mouse-grown NPC epithelial tumour cells of whatever ploidy do not clearly show a characteristic marker-chromosome change, and that the varied gross abnormalities of the near-triploid cells (HW, NM and LOL, Table II) do not present any consistent pattern. In addition, no apparent correlation has been seen with the present small series of tumours between the number of chromosome rearrangements and the origin of the material in question from either a primary or a secondary tumour (Tables I and II).

This work was assisted by the Cancer Research Campaign, London, England, out of funds donated by the Bradbury Investment Company, Hong Kong, and by the Stehlin Foundation for Cancer Research, Houston, Texas, USA. One of us (P.A.T.) was in receipt of a scholarship for training in research methods from the Medical Research Council, London.

### REFERENCES

Beatty-DeSana, J. W., Hoggard, M. J. & Cooledge, J. W. (1975) Non-random Occurrence of 7–14 Translocations in Human Lymphocyte Cultures. *Nature, Lond.*, **255**, 242.

de-Thé, G., Ambrosioni, J. C., Ho, H. C. & Kwan, H. C. (1969) Lymphoblastoid Transformation and Presence of Herpes-type Viral Particles in a Chinese Nasopharyngeal Tumour Cultured *in vitro*. *Nature, Lond.*, **221**, 770.

de-Thé, G., Ho, H. C., Kwan, H. C., Desgranges, C. & Favre, M. C. (1970) Nasopharyngeal Carcinoma. I. Types of Cultures Derived from Tumour Biopsies and Non-tumourous Tissues of Chinese Patients with Special Reference to Lymphoblastoid Transformation. *Int. J. Cancer*, **6**, 189.

Epstein, M. A. & Achong, B. G. (1977) Recent Progress in EB Virus Research. *Ann. Rev. Microbiol.*, **31**, 421.

Epstein, M. A., Achong, B. G. & Mansell, P. W. A. (1971) A New Virus in Cultures of Human Nasopharyngeal Carcinoma. In *Recent Advances in Human Tumor Virology and Immunology*. Ed. W. Nakahara, K. Nishioka, T. Hirayama and Y. Ito. Proc. 1st Int. Symp. Princess Takamatsu Cancer Res. Fund, pp. 163–171, Tokyo: University of Tokyo Press.

Fukuhara, S., Shirakawa, S. & Uchino, H. (1976) Specific Marker Chromosome 14 in Malignant Lymphomas. *Nature, Lond.*, **259**, 210.

Glaser, R., Nonoyama, M., Shows, T. B., Henle, G. & Henle, W. (1975) Epstein–Barr Virus: Studies on the Association of Virus Genome with Human Chromosomes in Hybrid Cells. *In: Oncogenesis and Herpesviruses II*, Lyon. IARC Scientific Publications No. 11, Vo. 1, pp. 457.

Hecht, F., McCaw, B. K., Peakman, D. & Robinson, A. (1975) Non-random Occurence of 7–14

Translocations in Human Lymphocyte Cultures. *Nature, Lond.*, **255**, 243.

JARVIS, J. E., BALL, G., RICKINSON, A. B. & EPSTEIN, M. A. (1974) Cytogenetic Studies on Human Lymphoblastoid Cell Lines from Burkitt's Lymphomas and Other Sources. *Int. J. Cancer*, **14**, 716.

JONDAL, M. & KLEIN, G. (1975) Classification of Lymphocytes in Nasopharyngeal Carcinoma (NPC) Biopsies. *Biomed.* **23**, 163.

KAISER-MCCAW, B., EPSTEIN, A. L., KAPLAN, H. S. & HECHT, F. (1977) Chromosome 14 Translocation in African and North American Burkitt's Lymphoma. *Int. J. Cancer*, **19**, 482.

KAISER-MCCAW, B., HECHT, F., HARNDEN, D. G. & TEPLITZ, R. L. (1975) Somatic Rearrangements of Chromosome 14 in Human Lymphocytes. *Proc. natn. Acad. Sci.* (*Wash.*), **72**, 2071.

KLEIN, G., GIOVANELLA, B. C., LINDAHL, T., FIALKOW, P. J., SINGH, S. & STEHLIN, J. S. (1974) Direct Evidence for the Presence of Epstein–Barr Virus DNA and Nuclear Antigen in Malignant Epithelial Cells from Patients with Poorly Differentiated Carcinoma of the Nasopharynx. *Proc. natn. Acad. Sci.* (*Wash.*), **71**, 4737.

MANOLOV, G. & MANOLOVA, Y. (1972) Marker Band in One Chromosome 14 from Burkitt Lymphomas. *Nature, Lond.*, **237**, 33.

NONOYAMA, M., HUANG, C. H., PAGANO, J. S., KLEIN, G. & SINGH, S. (1973) DNA of Epstein–Barr Virus Detected in Tissue of Burkitt's Lymphoma and Nasopharyngeal Carcinoma. *Proc. natn. Acad. Sci.* (*Wash.*), **70**, 3265.

PRIGOGINA, E. L. & FLEISCHMAN, E. W. (1975) Marker Chromosome 14q+ in Two Non-Burkitt Lymphomas. *Humangenetik*, **30**, 109.

REEDMAN, B. M. & KLEIN, G. (1973) Cellular Localization of an Epstein–Barr Virus (EBV)-associated Complement-fixing Antigen in Producer and Nonproducer Lymphoblastoid Cell Lines. *Int. J. Cancer*, **11**, 499.

SHANMUGARATNAM, K. (1971) Studies on the Etiology of Nasopharyngeal Carcinoma. *In*: *International Review of Experimental Pathology*, Vol. 10, Eds. G. W. Richter and M. A. Epstein. New York and London: Academic Press Inc. p. 361.

VISFELDT, J., POVLSEN, C. O. & RYGAARD, J. (1972) Chromosome Analyses of Human Tumours Following Heterotransplantation to the Mouse Mutant "Nude". *Acta. Path. Microbiol. Scand.*, Section A, **80**, 169.

WELCH, J. P. & LEE, C. L. Y. (1975) Non-random Occurrence of 7–14 Translocations in Human Lymphocyte cultures. *Nature, Lond.*, **255**, 241.

WOLF, H., ZUR HAUSEN, H. & BECKER, V. (1973) EB Viral Genomes in Epithelial Nasopharyngeal Carcinoma Cells. *Nature New Biol.* (*Lond.*), **244**, 245.

WURSTER-HILL, D. H., MCINTYRE, O. R., CORNWELL, G. G. & MAURER, L. H. (1973) Marker Chromosome 14 in Multiple Myeloma and Plasma Cell Leukaemia. *Lancet*, ii, 1031.

YATA, J., DESGRANGES, C., DE-THÉ, G., MOURALI, N., ELLOUZ, R., TACHIBANA, T. & BRUGERE, J. (1974) Nasopharyngeal Carcinoma VII. B and T Lymphocytes in the Circulating Blood and in Tumour Tissue. *Biomed.*, **21**, 244.

ZECH, L., HAGLUND, U., NILSSON, K. & KLEIN, G. (1976) Characteristic Chromosomal Abnormalities in Biopsies and Lymphoid Cell Lines from Patients with Burkitt and Non-Burkitt Lymphomas. *Int. J. Cancer*, **17**, 47.

ZUR HAUSEN, H., SCHULTE-HOLTHAUSEN, H., KLEIN, G., HENLE, W., HENLE, G., CLIFFORD, P. & SANTESSON, L. (1970) EBV DNA in Biopsies of Burkitt Tumours and Anaplastic Carcinomas of the Nasopharynx. *Nature, Lond.*, **228**, 1056.